# Organic nano-floating-gate transistor memory with metal nanoparticles

**DOI:** 10.1186/s40580-016-0069-7

**Published:** 2016-04-20

**Authors:** Luu Van Tho, Kang-Jun Baeg, Yong-Young Noh

**Affiliations:** 1 Department of Energy and Materials Engineering, Dongguk University, 30 Pildong-ro, 1-gil, Jung-gu, Seoul, 04620 Republic of Korea; 2 Department of Graphic Arts Information Engineering, Pukyong National University, 365 Sinseon-ro, Nam-gu, Busan, 48547 Republic of Korea

**Keywords:** Organic non-volatile memory, Metal nanoparticles, Nano-floating-gate, Organic field effect transistors

## Abstract

Organic non-volatile memory is advanced topics for various soft electronics applications as lightweight, low-cost, flexible, and printable solid-state data storage media. As a key building block, organic field-effect transistors (OFETs) with a nano-floating gate are widely used and promising structures to store digital information stably in a memory cell. Different types of nano-floating-gates and their various synthesis methods have been developed and applied to fabricate nanoparticle-based non-volatile memory devices. In this review, recent advances in the classes of nano-floating-gate OFET memory devices using metal nanoparticles as charge-trapping sites are briefly reviewed. Details of device fabrication, characterization, and operation mechanisms are reported based on recent research activities reported in the literature.

## Introduction

Organic electronic devices, such as organic field-effect transistors (OFETs) [1], organic light-emitting diodes [2], organic photovoltaic cells, and chemical and photo sensors [3, 4], have been developed as a result of intense research in both industrial and academic sectors. They have numerous advantages, such as the capability to realize flexible and large-area applications with low-cost and simple fabrication processes using graphic arts printing techniques [5–8]. Now the organic devices is a strong potential candidate for use in future soft electronics and wearable smart devices. Organic non-volatile memory (ONVM) is another important and fundamental element in the construction of electronic systems. Therefore, much work has been done towards developing high-density, low-cost, and non-volatile solid-state data storage devices [9–17]. Among the many different types of organic memory structures, OFET-based memory devices with a floating gate have been typically used because of their well-established operation mechanism, reliable memory operations, massive memory capacity, of stable charge storage for a sufficiently long time, which can easily fulfill the requirements for many targeted applications [18].

However, conventional floating-gate memory still has several limitations, such as difficulties in scaling down owing to increasing cell-to-cell interference, decreasing coupling ratio, and non-scalable tunneling oxide thickness owing to decreasing tolerance for charge loss [19–21]. In order to overcome those problems, recent research has focused on developing discrete charge trapping sites, including charge-trap dielectrics, such as silicon-oxide-nitride-oxide-silicon (SONOS) [22–25] and organic electrets [16] or nanocrystal (NC)-embedded dielectric layers, i.e., nano-floating-gate (NFG) memory devices with metal nanoparticles (NPs) and organic/inorganic nano-materials [26–28]. Compared to the conventional floating-gate cells, NFG memory generally shows better endurance, smaller chip size, multi-level capability, and lower power consumption; thus, this architecture of devices has become the leading technology in the state-of-the-art silicon-based flash memory industry. NFG memory devices using semiconducting or metallic NCs as a charge-trapping layer have advantages in that the charge-trap density (memory capacity) can be controlled by changing the size and spatial distribution of the NCs. Memory retention time also could be optimized by proper selection of metal NCs with deep work-function. Note that these parameters are important in determining the non-volatile memory characteristics, especially with regards to the programmed/erased bit distribution and data retention.

For enabling the organic NFG memory, controlled synthesis of semiconducting or metallic NCs is the first step, thereby incorporating those discrete trap sites inside of the gate dielectric layer. Recently, there have been many reports regarding the fabrication of NC-based memory devices utilizing metal nanoparticles, especially gold nanoparticles. In this article, recent studies and research activities are reviewed, mainly focusing on the fabrication and characterization of organic non-volatile memory devices with organic and inorganic nano-materials, including metal NPs, as the nano-floating-gate. OFET-based non-volatile memory devices gives a great opportunity for flexible, printable, and low-cost memory devices, since this NFG memory could be easily adopted in conventional flash memory technology.

## Floating-gate memory

**Fig. 1:**
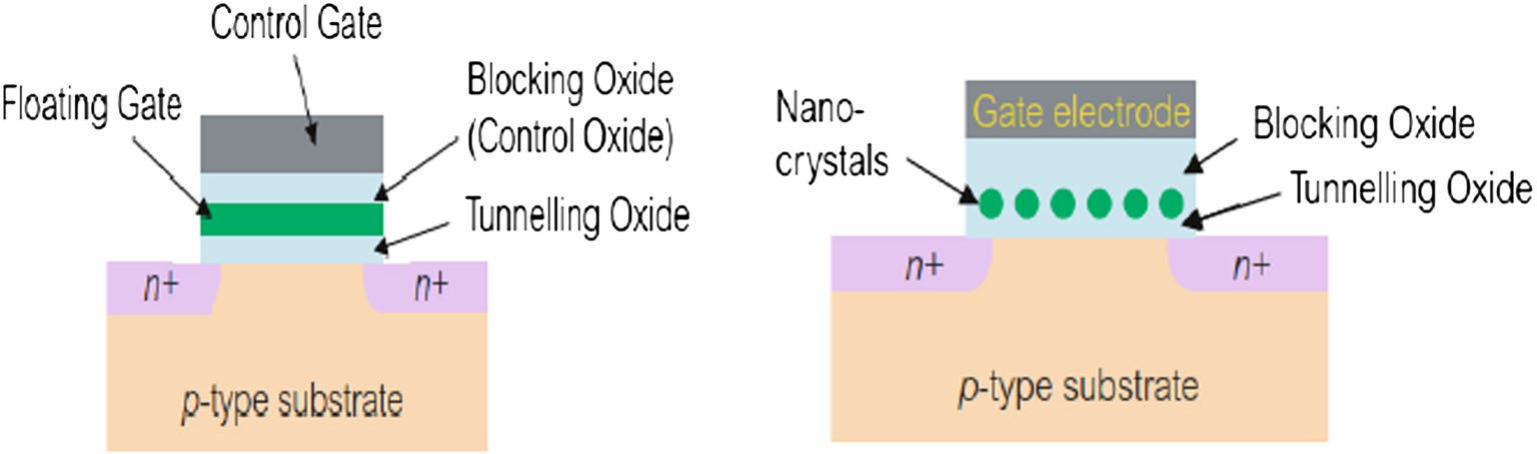
Schematic device structures of charge trapping floating gate (*left*) and nano-floating-gate memory devices (*right*). The figure is reproduced from Ref. [26] with permission, copyright 2010, IEEE

A floating-gate transistor has similar device structure to a regular OFET, except for the addition of the control gate, as shown in Fig. 1. The floating gate is embedded in the gate dielectric layer as a thin film or in discrete nanoparticle form [29]. OFETs normally operate in the accumulation mode; electrons or holes are induced by the application of an external gate electric field, negative or positive gate voltage (V_g_), respectively. This channel formation is ‘volatile’ because the accumulated charge carriers will be dissipated completely if no gate bias is applied. Therefore, in order to sustain those charge carriers in the channel region for non-volatile memory, the internal electric field has to be always loaded either by remnant dipoles of the ferroelectric materials or trapped charges inside of the dielectric layer.

**Fig. 2:**
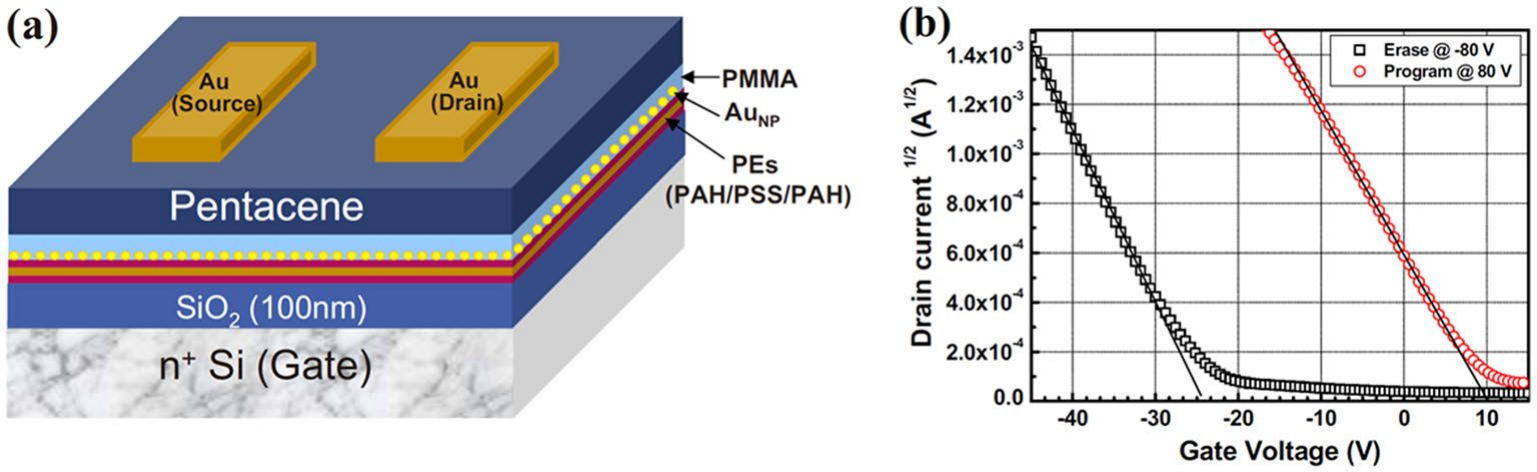
**a** Schematic device structure of typical *bottom*-gate and *top*-contact structured organic transistor-based nano-floating-gate memory devices. **b** Program/erase characteristics of the fabricated memory devices. The figure is reproduced from Ref. [41] with permission, copyright 2010, American Institute of Physics

**Fig. 3:**
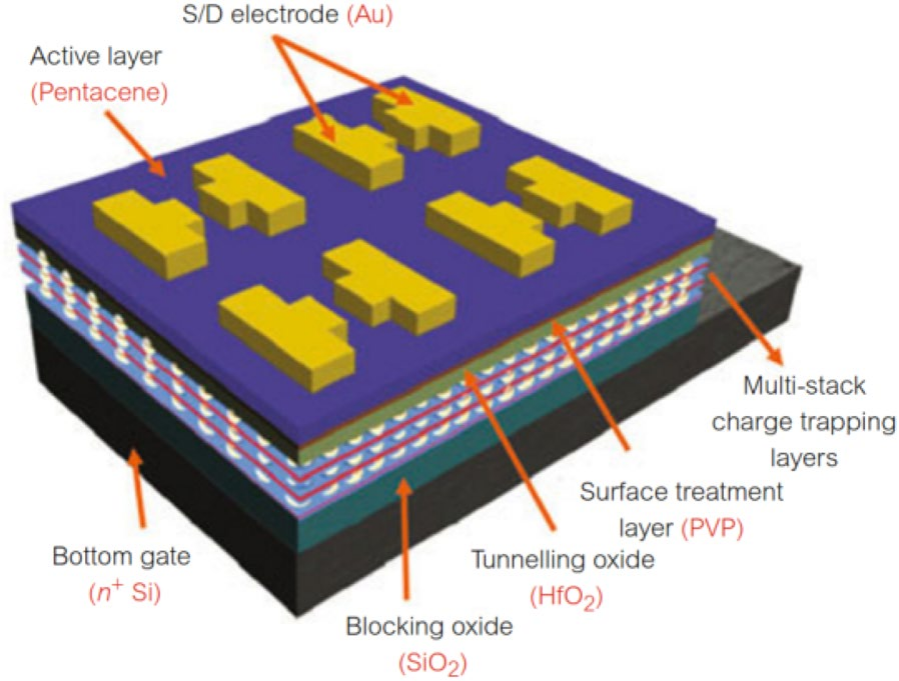
Schematic illustration of an organic transistor-based nano-floating-gate memory device with layer-by-layer assembled multi-stack charge trapping layers. The figure is reproduced from Ref. [30] with permission, copyright 2010, IEEE

The floating gate is completely separated by the dielectric layer, which consists of a thin tunneling layer and a robust charge-blocking layer between the semiconductor channel and the floating gate and the gate electrode and the floating gate, respectively. As shown device structures in Fig. 2a and Fig. 3. This memory structure is designed to increase the charging current of the floating gate and charge stored in the floating gate remains there without the need for any applied voltage (non-volatile memory) [30]. Electronic charge can be passed through the dielectric into the floating gate based on quantum tunneling or thermal emission during the programming process. Notably, in silicon-based flash memory technology, charge injection into the floating gate occurs typically by hot electron formation and Fowler–Nordheim tunneling mechanism. In contrast, direct band-to-band tunneling and thermal emission are more dominant charging mechanisms since hot electrons cannot be formed in the organic semiconductor channel. As can be seen in Fig. 2, charging the floating gate changes the threshold voltage (V_Th_ *)* of the transistor. The shift in the V_Th_ is proportional to the amount of charge located in the floating gate and inversely proportional to the dielectric capacitance. In the erasing memory process, an opposite gate voltage is applied, thus discharging the floating gate through the dielectric. In organic floating-gate memory, counter charge carriers are typically used to compensate the trapped charges in the floating gate.

To characterize an organic floating-gate memory, many parameters are used, such as the operating voltage, on/off ratio, memory window, program/erase speed, retention time, and endurance. First, the operating voltage of OFET memory is mainly determined by the minimum bias during programing and erasing processes to achieve a sufficient memory window and on/off current ratio. It is expected to be below 10 V because of the desire for low power consumption and to increase the reliability of the memory. Notably, organic floating-gate memory has a relatively high operation voltage above 50 V, since those reports were mostly focused on conceptual approaches and used conventional silicon dioxide or low permittivity (*k*) polymer gate dielectric layers with sufficient thickness. Therefore, the operating bias can be further decreased via use of high-*k* and thin gate dielectric layers.

The memory window (ΔV_Th_) is the most important parameter to distinguish the information storage level. It is defined by the V_Th_ shifted between the program and erase states. Because non-volatile memory devices have charge storage layers, the charge carriers can be stored (or trapped). From programming/erasing processes, the threshold voltage can be modulated since channel conductance is influenced by whether the charge carriers are stored in the floating gate or not. One can verify the programmed and erased states of the memory devices by measuring the drain current after application of programming and erasing bias, respectively. The difference in the drain current (i.e., on/off current ratio) can be measured in the range of memory window after programming and erasing processes [31–34].

The retention time is another important parameter for non-volatile memory. The stored charges are located in the floating gate and can be maintained even after the power is shut off, resulting in non-volatile memory operations. As time passes, charge stored in the floating gate will be diminished progressively through the leakage of current. For practical use in flash memory, the retention time is at least 10 years. However, organic floating-gate memory devices showed a retention time in the range of 10^6^ s, but this is still not enough and we need to find a way to keep the trapped charge carriers. It can be improved either by optimizing the device structure and circuit design, or formation of the floating gate, or by selection of a suitable dielectric layer. The endurance parameter is the ability of a memory device to withstand repeated program-read-erase cycles. If it is disposable memory, this endurance is not an important parameter. For most data storage media, however, the endurance is required to be at least 10^6^ cycles, which is seldom achieved for organic devices at present.

## Nano-floating-gate memory

Although conventional (continuous) floating-gate memory is the major technical advancement, there are many challenges as device scaling continues [35]. First, the tunneling layer thickness should be reduced, but itis very difficult to have a thin layer with sufficient charge retention and stress-induced leakage current characteristics. Other requirements also should be considered as the distance between layers becomes closer, such as the decrease of the coupling ratio owing to the increased parasitic capacitance and increased cell-to-cell interference. The scaling down of tunneling layer thickness can decrease the memory window margin as well as resulting in V_Th_ shift of memory cell [36]. Furthermore, with the smaller dimensions of floating gates, the amount of charge stored is reduced, resulting in very tight margins for the memory window. Therefore, many attempts have been made to solve these problems by inserting metal NPs into the storage layer. In this case, the problem originating from the use of continuous conducting floating gates can be effectively solved.

The memory devices based on metallic or semiconducting NPs have many advantages compared to the conventional floating gate. First, because the NPs are deposited on the tunneling dielectric layer discretely, there is no effect on the conducting floating gate layer. Trapped charge carriers in discrete NPs can be retained for a long time. Second, the charge storage levels and trap sites can be effectively controlled by manipulating the materials, size, and density of the NPs. Therefore, many efforts have been made toward the development of nanocrystal-based non-volatile memory devices.

## Recent advance in nano-floating-gate memory

Among the various species of metal NPs, gold NPs are widely used in NFGs owing to relatively easy synthesis, chemical and operational stability, and deep potential wall from its high work function. In 2006, Liu et al. [37] introduced an OFET memory with self-assembled gold NPs embedded in the gate dielectric. The transistor-based memory was fabricated on an n-type silicon substrate containing a silicon oxide (SiO_2_) layer with a thickness of 100 nm, where silicon serves as the control gate electrode and the SiO_2_ layer as the charge-blocking dielectric. The discrete gold NPs were deposited onto the oxide surface by electrostatic layer-by-layer self-assembly method. Solution-processed poly(3-hexylthiophene) (P3HT) is used as a semiconductor channel layer, which is separated by additional polyelectrolytes and a poly(4-vinylphenol) (PVP) tunneling layer that covered the gold NPs. The memory transistor had a sufficient on/off current ratio of over 1500, but showed a short data retention time of about 200 s. In 2010, gold NPs were synthesized by Ostwald ripening and chemical solution methods, and applied in a memory device [38]. The charge trapping layer was fabricated mostly by self-assembly processes, depositing very thing old layers on the oxide-covered silicon substrates, then post-annealing process to form gold NPs by Ostwald ripening. In this effect, a thin gold layer was converted to gold nanoparticles owing to the minimization of the surface energy [39]. Lee et al. [40] reported non-volatile transistor memory using the self-assembled gold nanoparticles method. A very thin tunneling oxide layer (~3 nm) and a gold layer (~1.2 nm)were first formed and thermally annealed at 575 °C in order to form the discrete gold NPs layer. Those thin dielectric layers enabled NFG memory with a large memory window under low programing and erasing bias conditions.

In 2010, Kim et al. [41] used poly (methyl methacrylate) (PMMA) in place of oxides as a tunneling dielectric layer (see Fig. 2) . They demonstrated a maximum memory window of 34 V with a programming voltage of 80 V. Notably, the data retention time could be remarkably improved to more than 1 year. They also further demonstrated multi-layered gold NPs charge trapping elements via layer-by-layer self-assembled method in order to enlarge the memory window [42]. The results showed that the memory window was increased from 11 to 14.6 V, corresponding to increasing the number of charge trapping layers from two to three.

Zhen et al. [43] reported an organic NFG memory device with a memory window of 20 V, which was based on copper (II) phthalocyanine (CuPc) thin-film transistors using a polyimide gate dielectric layer containing embedded gold NPs. For achieving small programing and erasing biases, Chang et al. [44] used high-k oxide dielectrics, such as HfLaO (20 nm), HfON (20 nm), and HfO (6 nm) as the blocking, charge trapping, and tunneling dielectric layers, respectively. Their memory devices exhibited a low program/erase voltage of 12 V, a fast programming speed of 1/100 ms, a sufficient memory window of 2.4 V, and stable charge retention of its initial memory window to 0.78 V even after 48 h. In 2009, Sekitani et al. [45] developed a low power operating flexible floating gate transistor memory using self-assembled molecular gate insulators, which enabled very small program and erase voltages of –6 to +3 V. It was one of the best results reported to date for organic flash memory. The floating gate was a 20-nm thick evaporated aluminum layer, the top and bottom dielectrics were both a combination of a 4-nm thick aluminum oxide (AlO_*x*_) layer with a 2-nm thick alkyl phosphonic acid self-assembled monolayer.

**Fig. 4:**
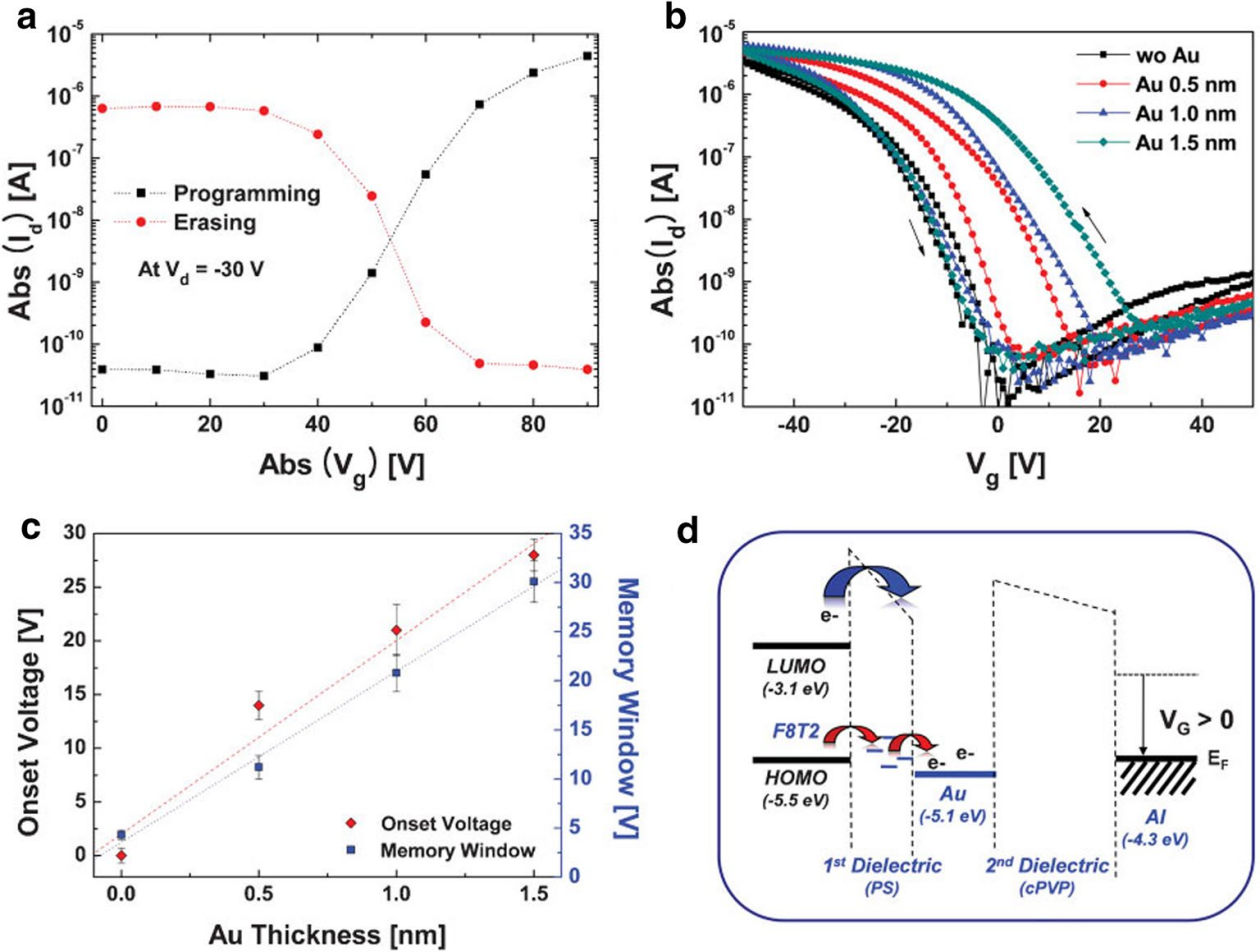
Controllable shifts in the threshold voltage of *top*-gated polymer transistors using Au nano-crystals. **a** Programming and erasing characteristics of F8T2 FET memory devices. **b** Transfer characteristics of F8T2 FET devices after thermal evaporation of different thicknesses of Au, from 0.5 to 1.5 nm. **c** Change of onset voltages and the amount of memory window for polymer transistor memory. **d** Schematic demonstration of a potential operation mechanism of floating-gate F8T2 FET memory devices. The figure is reproduced from Ref. [47] with permission, copyright 2010, WILEY–VCH Verlag

Staggered top-gate OFET structure provides significant advantages over other structures, such as auto encapsulation of an air- and/or photo-sensitive active channel and reduction of the contact resistance. In 2009, Wang et al. [46] found a memory effect based on the top-gate configuration by inserting a layer of nanoparticles, such as silver (Ag) or calcium fluoride (CaF_2_), as the floating gate between two Nylon 6 gate dielectrics. Baeg et al. also achieved a top-gate OFET memory with poly[9,9-dioctylfluorenyl-2,7-diyl]-co-(bithiophene)] (F8T2) polymer semiconductor with good performance by embedding gold NPs between the bi-layered polymer gate dielectrics, polystyrene (PS) and cross-linked PVP layers, which were used as charge injection and current blocking gate dielectrics, respectively [47] (see Fig. 4). Reversible control of the V_Th_ and reliable memory characteristics was achieved by the incorporation of thin Au NPs as charge storage sites for negative charges (electrons).

**Fig. 5:**
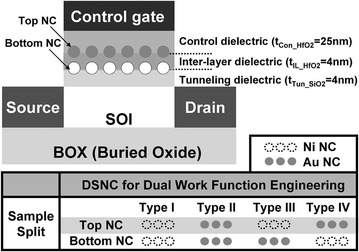
Designed work-function engineering of double-stacked metal nanocrystals for non-volatile memory application. The figure is reproduced from Ref. [48] with permission, copyright 2009, IEEE

Ryu et al. [48] used double-stacked metal NP layers to fabricate non-volatile transistor memory (see Fig. 5). They deposited different sequences of gold and nickel nanoparticle charge trapping layers (i.e., Ni/Ni, Au/Au, Ni/Au, and Au/Ni) and verified that the higher and lower work function metal nanoparticles resulted from combinations of top and bottom charge trapping layers (Au/Ni) could provide fast program/erase speeds and a long retention time.

**Fig. 6:**
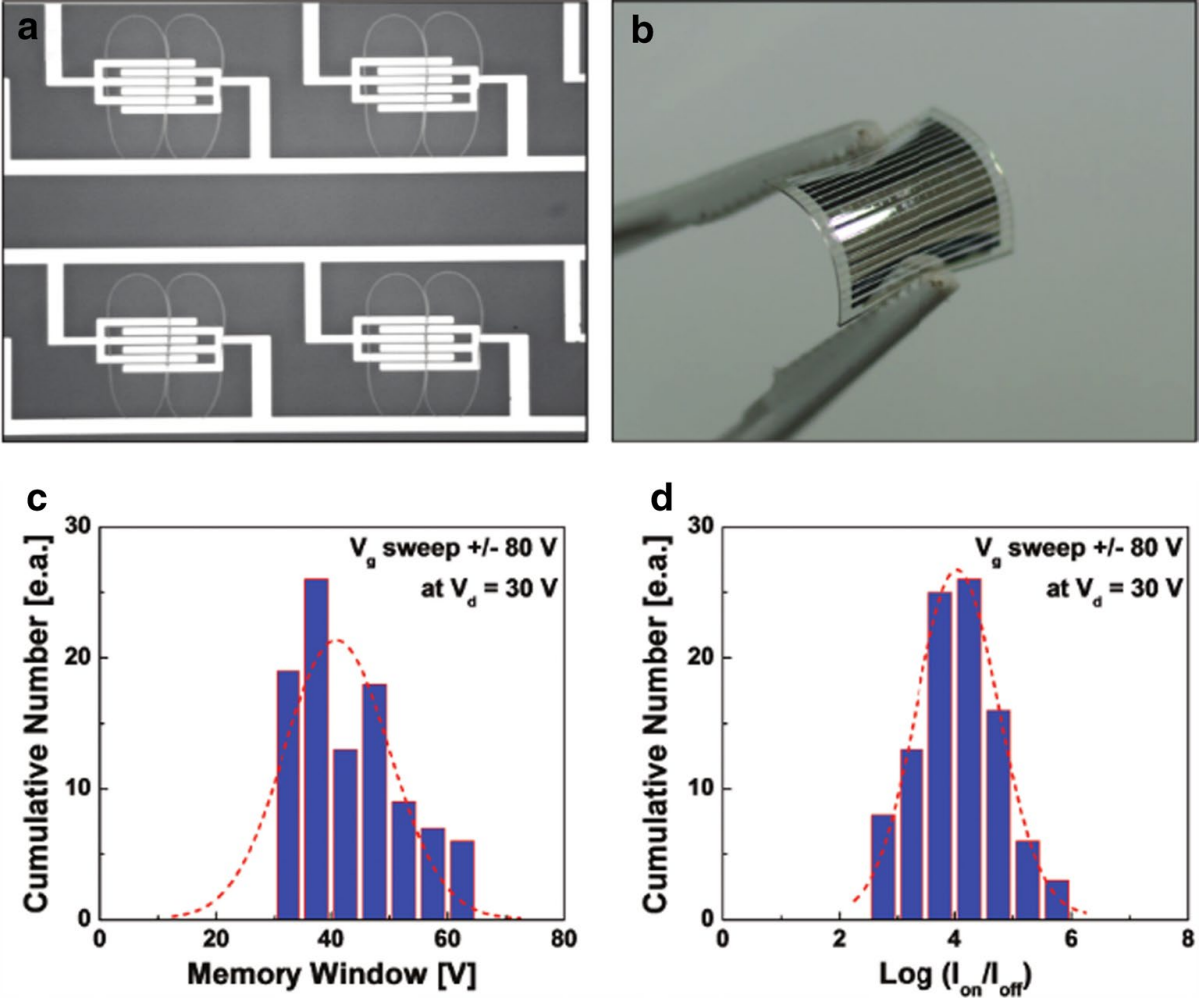
Inkjet-printed and flexible organic nano floating gate memory arrays (16 × 16 organic field-effect transistors, 256 bit OFET memory array). **a** A digital camera image of the active feature of an inkjet-printed P(NDI2OD-T2)-based device; **b** digital image of the corresponding 256-bit OFETs memory array on a flexible polyethylene naphthalate (PEN) substrate. Frequency distributions of the measured values of the **c** memory window and **d** log(I_on_/I_off_) at V_d_ = 30 V of the devices in the 256-bit memory array on a PEN flexible substrate. The figure is reproduced from Ref. [49] with permission, Copyright 2013 WILEY–VCH Verlag

Kang et al. [49] reported high performance organic NFG memory devices using various blocking dielectric layer and metal NPs as charge-trapping site structures. They were fabricated from the n-type polymer semiconductor, poly{[*N*,*N*’-bis(2-octyldodecyl)-naphthalene-1,4,5,8-bis(dicarboximide)-2,6-diyl]-alt-5,5-(2,2′bithiophene)}[P(NDI2OD-T2)], and different metal nanocrystals, such as gold (Au), silver (Ag), copper (Cu), and aluminum (Al). These NPs were embedded within the bilayers of various polymer dielectrics, PS/PVP and PS/PMMA. The P (NDI2OD-T2)OFET-based NFG memory devices exhibited high electron mobilities of 0.4–0.5 cm^2^ V^−1^s^−1^ and very reliable non-volatile memory characteristics; a wide memory window of ~52 V, high on/off current ratio of ~10^5^, and a long extrapolated retention time more than 10^7^ s. Those results were strongly dependent on the choice of the blocking dielectric (PVP or PMMA) and the metal (Au, Ag, Cu, or Al) nanocrystals. Notably, the best memory characteristics were achieved in the devices fabricated using PMMA and Au or Ag NPs. The polymeric NFG memory devices with PMMA and spatially well-distributed Cu NPs showed quasi-permanent retention characteristics. As shown in Fig. 6, based on the inkjet-printed P (NDI2ODT2) semiconductor, they developed a 256-bit printed and flexible organic NFG memory array consisting of 16 × 16 transistors with Au-NPs on a polyethylenenaphthalate substrate. These memory devices in array exhibited a high on/off current ratio (~10^4±0.85^), wide memory window (~43.5 ± 8.3 V), and a high degree of reliability.

## Summary and outlook

Major research has been reviewed on the fabrication and characterization of organic NFG memory devices based on metal NPs. Noble metal NPs, such as gold and silver, have been mostly used as the charge trapping elements because of their relatively simple synthesis, stability, and high work function. Non-volatile memory characteristics are strongly dependent on different metal species, and the size and spatial distribution of the discrete nano-sized trap sites. Thus, controllability is another advantage of NFG memory. In addition, various methods were successfully performed to enhance the electrically programmable memory characteristics. However, these research non-volatile memory devices based on metal nanoparticle are still limited to the fabrication and characterization of unit devices. Product-level device applications are challenging for the next generation of printed and flexible solid-state data storage.
